# New Insights into Conformationally Restricted Carbonic Anhydrase Inhibitors

**DOI:** 10.3390/molecules28020890

**Published:** 2023-01-16

**Authors:** Jacob Combs, Murat Bozdag, Lochlin D. Cravey, Anusha Kota, Robert McKenna, Andrea Angeli, Fabrizio Carta, Claudiu T. Supuran

**Affiliations:** 1 Department of Biochemistry and Molecular Biology, College of Medicine, University of Florida, Gainesville, FL 32610, USA; 2 NEUROFARBA Department, Sezione di Scienze Farmaceutiche e Nutraceutiche, University of Florence, Via Ugo Schiff 6, 50019 Florence, Italy

**Keywords:** X-ray crystallography, carbonic anhydrase IX, structure activity relationships

## Abstract

This paper reports an investigation into the impact of pyridyl functional groups in conjunction with hydroxide-substituted benzenesulfonamides on the inhibition of human carbonic anhydrase (CA; EC 4.2.1.1) enzymes. These compounds were tested in vitro of CA II and CA IX, two physiologically important CA isoforms. The most potent inhibitory molecules against CA IX, **3g**, **3h**, and **3k**, were studied to understand their binding modes via X-ray crystallography in adduct with CA II and CA IX-mimic. This research further adds to the field of CA inhibitors to better understand ligand selectivity between isoforms found in humans.

## 1. Introduction

Precision medicine is typically understood as exclusive targeting between closely related enzymatic types/isoforms within the field of medicinal chemistry [1,2,3]. Small molecules can be used as a type of precision medicine; however, they typically do not have high selectivity for their biological target if it exists in a similar structure or function to other enzymes/receptors. Human Carbonic Anhydrases (CAs, EC 4.2.1.1) catalyze the reversible conversion of carbon dioxide and water into bicarbonate and a proton via a coordinated metal ion, typically zinc [4]. CA II expression is the most widespread in tissue types [5]. CAs dysregulation is linked to many diseases, including cancers expressing CA IX [6,7,8,9]. This upregulation of CA IX expression allows cancer to have a selective advantage in the hypoxia-induced acidic microenvironment, causing an increase in tumor aggression [10,11]. As such, the development of CA inhibitors (CAIs) has been a common topic of study. With CA isoforms sharing similar structural features, designing CAIs to specifically target a single isoform has proved challenging. This study examined modes of binding between different compound scaffolds oriented in the active site of CA isoforms. These scaffolds of interest include pyridyl conjugated to the well-known benzenesulfonamide inhibitors of CA. The inclusion of the pyridyl group increased specificity of CA IX over CA II as possible molecules to be used for the treatment of cancer.

## 2. Results and Discussion

### 2.1. Chemistry

**Scheme 1:**
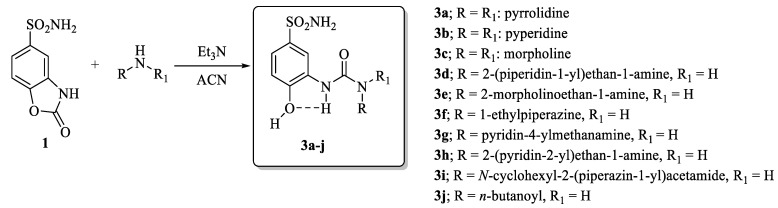
Synthesis of conformationally restricted CAIs **3a**–**j**.

The synthetic strategy adopted for the synthesis of the compounds reported in this manuscript was first introduced by researchers with the intent to decipher the details of the contribution onto CA isoform selectivity given from specific fragments connected to the ureido group in depth [12]. Such an approach became of large experimental applicability when the synthesis of compounds of the type in Scheme 1 bearing alkyl ureas endowed with differential rotational properties were accessed by means of nucleophilic attack of selected primary/secondary amines on the ad hoc prepared 2-oxo-2,3-dihydrobenzo[*d*]oxazole-5-sulfonamide **1** [1,12,13]. By making use of the structure activity relationship (SAR) knowledge acquired in the previous reports, herein we introduce ad hoc moieties able to afford highly potent and selective ligands for the tumor associated CA IX isoform (Scheme 1).

**Scheme 2:**
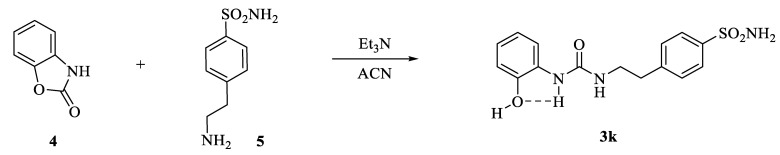
Synthesis of conformationally restricted CAI **3k**.

The newly introduced compound, namely **3k** in Scheme 2, was sought to introduce conformational restrictions at the tail end of the CAI using the same principle of the aforementioned strategy.

The compound **3k** was synthesized through the addition of commercially available compounds **4** and **5** under the same conditions set for the derivatives **3a**–**j**. The formation of the intramolecular and conformational locking five-membered ring in **3k** was experimentally observed by means of the ^1^H-NMR technique, which accounted for a phenolic singlet at 9.82 ppm. All compounds reported here were purified by silica gel column chromatography or crystallization from the appropriate solvents and were fully characterized by means of ^1^H-NMR, ^13^C NMR and mass spectra (ESI-MS).

### 2.2. In Vitro Carbonic Anhydrase Inhibition

All compounds reported in this work were investigated for their ability to physiologically inhibit the most relevant CA isoforms I, II, IX, and XII. The inhibition properties of **3a**–**k** were all assessed in vitro by means of the stopped-flow technique and compared to the standard CAI of the sulfonamide type acetazolamide (**AAZ**). The obtained activities in Table 1 were all reported as K_I_ values.

The above reported data (Table 1) allowed for the deciphering of the SARs governing the potency and selectivity of **3a**–**k** on CA isozymes.

Among the cyclic ureido derivatives, the pyrrolidine **3a** resulted in the most effective CA I inhibitors (K_I_ of 583.1 nM) because ring expansion (**3b**) or introduction of a heteroatom (**3c**) spoiled the inhibition potency up to 14.8- and 6.9-fold respectively. The K_I_ values associated with the monosubstituted ureas **3d**–**k** evaluated as inhibitors of the isoform I were highly variable For instance, the pyperidin-1-yl ethane **3d** was a high nanomolar inhibitor (i.e., K_I_ of 688.6 nM). Substitution at 4-position of the cycloalkyl of the *sp*^3^-carbon with isostructural oxygen and nitrogen, as in **3e** and **3f**, diminished the ligands affinities with the same magnitude (i.e., 13.4- and 13.1-fold respectively). A slight potency increase (i.e., 1.2-fold) was observed when the piperidinyl group in **3d** was replaced with the 2-pyridine moiety instead to afford **3h** (K_I_ of 571 nM). Quite interestingly, the compound **3g** in which the 4-methylen substituted pyridine resulted in the most effective CA I inhibitors among the series and was up to 2.8-fold more potent when compared to the standard **AAZ**. Conversely, insertion of alkyl tails such as the *n*-butanoyl in **3j**, the esaprazole in **3i**, or the tail-locked moiety **3k** did not result in appreciable inhibition of CA I (Table 1).

The set of compounds reported were also not particularly effective in inhibiting CA II because the K_I_ associated values were all in the high nano-low micromolar range and were thus far from the **AAZ** K_I_ of 12.1 nM (Table 1). SAR considerations allowed for the determination that the kinetic trend of the compound series on CA II was not related to the previously discussed isoform I. For instance, the piperidine derivative **3b** was more effective (i.e., 3.5-fold) than its restricted pyrrolidine counterpart **3a**, and the morpholine tail (as in **3c**) was very detrimental for the ligand interaction being the less effective CA II inhibitor among all the compounds reported (K_I_ of 8026 nM). The introduction of cycloalkyl tails by means of a linear ethyl linker (derivatives **3d**–**f**) showed that the presence of a *sp*^3^-carbon atom (**3d**) was far more tolerated over an oxygen (**3e**) or nitrogen atom (**3f**) as the associated experimental K_I_ values were progressively increased (K_I_ of 287.8, 2995, and 7741 nM for **3d**, **3e**, and **3f**, respectively). Simple *n*-alkyl tails such as in **3j** or structurally more complex ones such as the esaprazole in **3i** afforded medium nanomolar inhibition values (K_I_s of 280 and 405.4 nM) with the former being more closely matched to **3d** (Table 1). Similarly, the pyridine substituted derivatives **3g** and **3h** gave each other close K_I_ values of 463 and 331 nM, respectively. Interestingly, the newly introduced **3k** was the best performing CA II inhibitor on the list with a K_I_ of 75.9 nM, although it 6.3-fold less effective than **AAZ**.

Better defined SARs were obtained when the tumor associated CA IX was considered (Table 1). Although compounds **3a** and **3b** were almost equipotent inhibitors of the IX isoform, with the latter only 1.2-fold more effective (K_I_s of 369.1 and 307.9 nM), the introduction in **3b** of an oxygen atom at 4-position instead, to afford the morpholine containing derivative **3c**, induced a 2.2-fold potency increase (K_I_ of 139.8 nM). Identical kinetic patterns were also observed in **3d** and **3e**, which bear—by means of an ethyl tether—a piperidine and morpholine terminal moiety, respectively (K_I_s of 4116 and 2142 nM). In opposition to the cytosolic expressed CAs I and II, the IX isoform **3e** resulted in 1.9-fold more potent inhibition when compared to **3d**, and the piperazine **3f** showed equal potency to the former (K_I_s of 2142 and 2153 nM for **3e** and **3f**, respectively). Medium nanomolar potency accounted for either the esaprazole **3i** (K_I_ of 204.2 nM) or the *n*-butanol **3j** (K_I_ of 215 nM) derivatives. The introduction of the phenolic terminal moiety as in **3k** was also beneficial to the inhibition potency, which showed a K_I_ value of 34.7 nM and was thus comparable to the reference **AAZ** (K_I_ of 25.8 nM). Of note, the pyridine containing derivatives **3g** and **3h**, which reported sub-nanomolar K_I_ values of 0.30 and 0.24 nM, respectively, were thus the most potent CA IX inhibitors among the compounds reported.

As for the remaining tumor associated CA XII isoform, enhanced discrimination was observed among **3a** and its ring expanded the counterpart **3b** with the former being a 5.5-fold more effective inhibitor (K_I_s of 91.3 and 506.6 nM for **3a** and **3b** respectively). Moreover, the introduction of the morpholine moiety, such as in **3c**, further lowered the K_I_ value up to 73.8 nM. The insertion of an ethyl spacer between the ureido moiety, and either the piperidine or morpholine rings to afford **3d** and **3e**, respectively, flattened the kinetic profile (K_I_s of 659.1 and 628.8 nM for **3d** and **3e**, respectively). The same effect was also reported for the piperazine **3f**, which was only a 1.3-fold more effective CA XII inhibitor when compared to the least potent **3d**. In analogy to CA IX, the installation of aromatic tails such the substituted pyridines in **3g** and **3h** were also beneficial for inhibition of the CA XII isoform (K_I_s of 85.1 and 65.9 nM for **3g** and **3h**, respectively). The effects on kinetic values of the *n*-butanoyl (i.e., **3j**) and esaprazoyl (i.e., **3i**) tails were of particular interest. As reported in Table 1, the derivative **3j** showed an experimental K_I_ value of 47.1 nM, which was even lower (i.e., 33.7 nM) for **3i**. Conversely, the phenolic-containing terminal moiety **3k** resulted in a medium nanomolar CA XII inhibitor (K_I_ of 238.6 nM). 

Overall, the results reported in Table 1 allowed us to select highly effective CA IX inhibitors with sub-nanomolar K_I_ values and endow them with elevated isoform selectivity for the off-target house-keeping CA II isoform. 

### 2.3. X-ray Crystallography

X-ray crystallography was used to determine the structures of both CA II and CA IX-mimic in the complex with three inhibitor compounds (**3g**, **3h**, and **3j**). These compounds are identical in the zinc-binding group composed of a multiply substituted benzenesulfonamide, with a *para*-substituted hydroxide and an *ortho*-substituted ureido-linker group attaching the head to the tail group, which is the variable region in this experiment. A naming convention similar to the one utilized in Bozdag et al., 2022 [12] is present in this work as well; however, the substitution pattern relative to the *para*-hydroxide is signified by the first character, the functional group by the second, and the number of carbons linking the two is the final digit [12].

**Figure 1:**
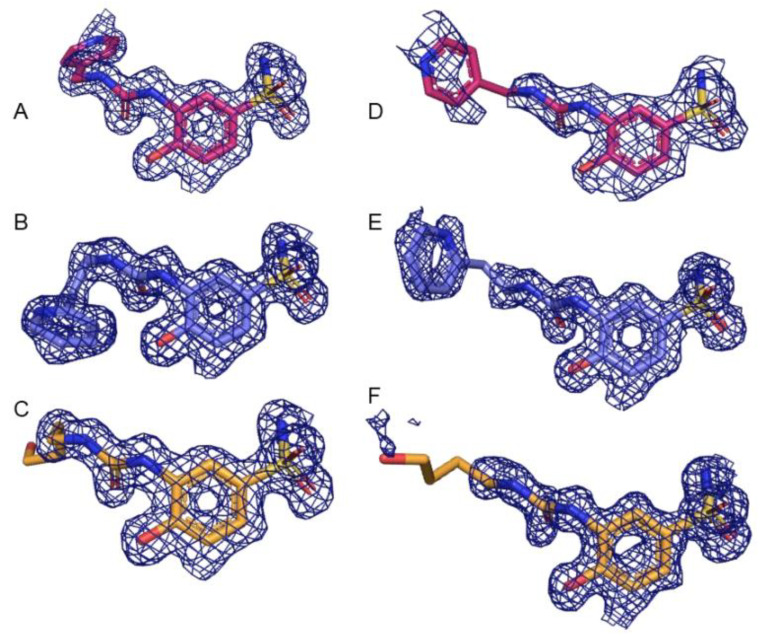
Electron density (blue mesh) of each complex at 1.0 σ. Compound **3g** is shown in pink, compound **3h** is shown in purple, and compound **3j** is shown in orange. (**A**) Compound **3g** bound to CA II, (**B**) **3h** bound to CA II, (**C**) **3j** bound to CA II, (**D**) **3g** bound to CA IX-mimic, (**E**) **3h** bound to CA IX-mimic, and (**F**) **3j** bound to CA IX-mimic.

Diffraction data was collected on the F1 beamline at Cornell High Energy Synchrotron Source (CHESS) and Stanford Synchrotron Radiation Lightsource (SSRL). All solved structures had a resolution range between 1.1 Å (CA IX-mimic and **3j**) and 2.0 Å (CA IX-mimic and **3g**) and were resolved to space group P 1 2_1_ 1. All complexes had between 30,931 (CA IX-mimic and **3g**) and 279,296 reflections (CA IX-mimic and **3j**), redundancy between 1.9 (CA IX-mimic and **3g**) and 3.4 (CA II and **3g**), and overall completeness between 92% (CA IX-mimic and **3j**) and 100% (CA IX-mimic and **3h**). There were no Ramachandran outliers, and at least 96% of the φ-ψ angles for the residues in every structure were in Ramachandran-favored regions. Mean I/σ ranged from 7.6 (CA IX-mimic and **3g**) to 18.6 (CA II and **3j**) and average temperature factors ranged from 18.3 Å^2^ (CA IX-mimic and **3h**) to 27.4 Å^2^ (CA IX-mimic and **3g**) (Table 2). Electron density of each ligand in complex with CA II and CA IX at 1.0 σ is well-fitted and corroborates the B-factor statistics (Figure 1). Additionally, these structures were deposited in the Protein Data Base (PDB) with the following PDB excision codes: 8FQX (CA II + **3g**), 8FQY (CA II + **3h**), 8FQZ (CA II + **3j**), 8FR1 (CA IX-mimic + **3g**), 8FR2 (CA IX-mimic + **3h**), and 8FR4 (CA IX-mimic + **3j**).

**Figure 2:**
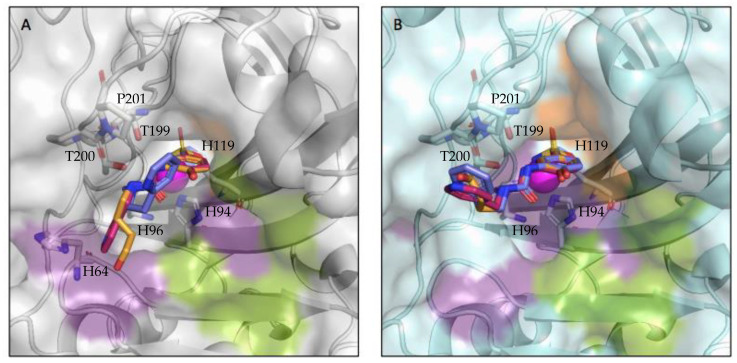
Compounds bound to the active site of (**A**) CA II (grey) and (**B**) CA IX-mimic (cyan) with the hydrophobic (orange), hydrophilic (purple), and selective (green) pockets shown. Zinc depicted as a magenta sphere, compound **3g** is shown in pink, compound **3h** is shown in purple, and compound **3j** is shown in orange; the three compounds have been overlayed.

Binding to the active site of both CA II and CA IX-mimic was observed in crystallographic experiments (Figure 1). Interestingly, there was little variability in the active site interactions observed as all but one protein–ligand complex was bound to the same three residues. All three compounds interacted with the left side of the pocket leading to the active site, while the primary differences observed were in the tail group conformation (Figure 2). All compounds formed hydrogen bonds with the same three residues, T199, T200, and P201, that are conserved between CA II and CA IX-mimic. T199 generally was bound to the sulfonamide head, along with the centrally coordinated catalytic zinc ion. T200 formed hydrogen bonds with the nitrogen that linked the benzene and the ureido group. P201 formed hydrogen bonds with the nitrogen that linked the ureido group to the alkyl linker group. Notably, **3g** was observed to share a hydrogen bond with H64 in complex with CA II, an interaction not observed when binding to CA IX-mimic or by any other ligand binding to any other complex. 

**Figure 3:**
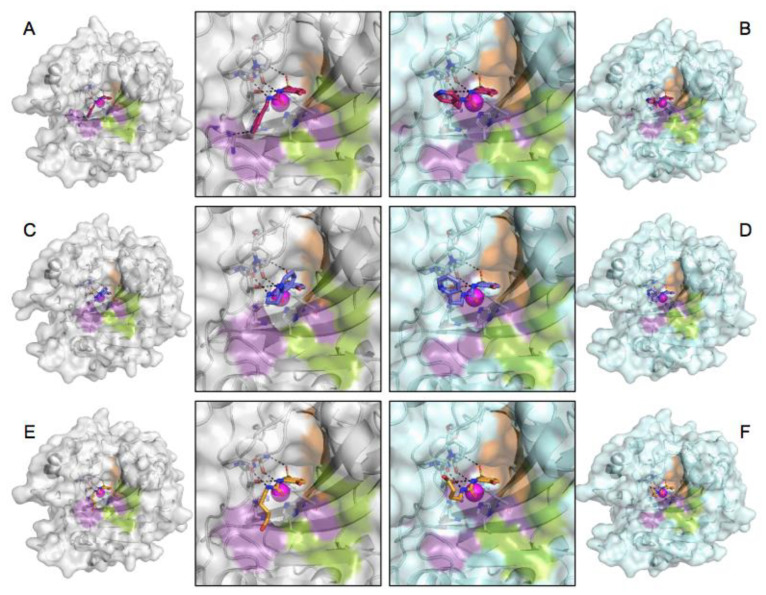
CA-compound complex, the complete structure and close-up of the active site. (**A**) Compound **3g** bound to CA II, (**B**) **3g** bound to CA IX-mimic, (**C**) **3h** bound to CA II, (**D**) **3h** bound to CA IX-mimic, (**E**) **3j** bound to CA II, and (**F**) **3j** bound to CA IX-mimic. CA II (grey) and CA IX-mimic (cyan) with the hydrophobic (orange), hydrophilic (purple), and selective (green) pockets shown. Zinc depicted as a magenta sphere, hydrogen bonds as black dashes, compound **3g** is shown in pink, compound **3h** is shown in purple, and compound **3j** is shown in orange.

Compounds **3g** and **3h** have highly similar structures, similar binding patterns to CA IX-mimic, and similar affinity for CA IX over CA II. Compound **3g** contains a 2-ureidobenzenesulfonamide and **3h** contains a 1-ureido-benzenesulfonamide, relative to the heterocyclic pyridyl functional group. The pyridyl group is at a 107° angle (-NH-CH2-Py) in **3g** and a 114° (-NH-CH2-CH2) angle for **3h**, highlighting the similarity between the two high affinity complexes that exemplify a textbook case of isozyme-specific selectivity. Both **3g** and **3h** have a hydrophobic interaction with F131 in complex with CA II, and this bulky residue introduces energetically disfavored steric hindrance in its interaction with the *para*-hydroxide of the benzenesulfonamide zinc-binding group. This hydroxide is rigidly set in its position, roughly 3.0 Å away from F131, as a result of the tight < 2 Å bond formed between the sulfonamide and zinc and the planarity of the benzenesulfonamide. This disfavored polar-hydrophobic interaction is somewhat mitigated in the complex formed between **3g** and CA II, by way of a hydrogen bond formed between H64 and the pyridine ring. The substitution of the pyridine and slightly elongated (one more carbon) chain of **3h** are most likely responsible for the absence of this interaction (Figure 3).

Both **3g** and **3h** have poor binding affinity to CA II as a result of steric hindrance of F131 on the *para*-hydroxide of the benzenesulfonamide driving the tail group to the hydrophilic site that is weakly stabilized by way of hydrogen bonding and weak electrostatic interactions between hydrophilic pocket residues and the ureido linker as well as the terminal pyridine (Figure 3). The conformational freedom afforded by F131V in CA IX-mimic abates the energetic penalty of this polar–hydrophobic interaction and allows for the pyridine to maximize the much-preferred hydrophobic interactions between the carbon chain of the linker. Compound **3j** was both weakly selective and had a similarly poor affinity for both CA II and CA IX at 280 nM and 215 nM, respectively (Table 1). This is likely due to a similar phenomenon observed and discussed in our previous study [12]. The ureido-butyl linker-tail group is meta-substituted on the benzenesulfonamide and the substitution pattern on the benzenesulfonamide has been previously observed to have a steering effect on the tail region as a result of the rigid plane of the benzenesulfonamide. The lipophilic tail of **3j** is in energetically unfavorable positions in complex with both CA II and CA IX-mimic, where it is situated in the hydrophilic pocket in dramatically disparate tail conformations. Compound **3j**’s affinity profile and binding conformation illustrate the imperative nature of targeting the selective pocket while **3g** and **3h** illustrate the profound impact on binding affinity that can be conferred by designing drugs that effectively leverage this site (Figure 3).

## 3. Experimental Section

### 3.1. Chemistry

**Scheme 3:**
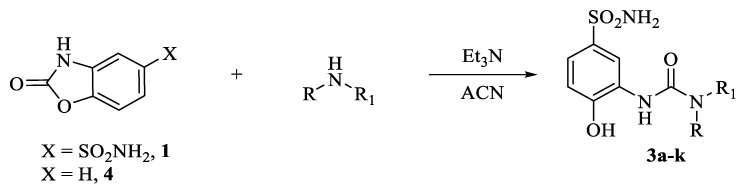
Synthesis of 3**a**–**k**: General procedure to synthesize reported compounds 3**a**–**k**.

Anhydrous solvents and all reagents were purchased from Sigma-Aldrich, VWR, and TCI. All reactions involving air- or moisture-sensitive compounds were performed under a nitrogen atmosphere. Nuclear magnetic resonance (^1^H NMR, ^13^C NMR, ^19^F NMR) spectra were recorded using a Bruker Advance III 400 MHz spectrometer in DMSO-*d_6_*. Chemical shifts are reported in parts per million (ppm) and the coupling constants (*J*) are expressed in Hertz (Hz). Splitting patterns are designated as follows: s, singlet; d, doublet; t, triplet; m, multiplet; brs, broad singlet; dd, double of doubles. The assignment of exchangeable protons (N*H*) was confirmed by the addition of D_2_O. Analytical thin-layer chromatography (TLC) was carried out on Merck silica gel F-254 plates. Flash chromatography purifications were performed on Merck silica gel 60 (230–400 mesh ASTM) as the stationary phase, and ethyl acetate, *n*-hexane, acetonitrile, and methanol were used as eluents. The solvents used in MS measurements were acetone and acetonitrile (Chromasolv grade) purchased from Sigma-Aldrich (Milan, Italy), and mQ water 18 MΩ obtained from Millipore’s Simplicity system (Milan, Italy). The mass spectra were obtained using a Varian 1200L triple quadrupole system (Palo Alto, CA, USA) equipped with electrospray source (ESI) operating in both positive and negative ions. Stock solutions of analytes were prepared in acetone at 1.0 mg mL^−1^ and stored at 4 °C. Working solutions of each analyte were freshly prepared by diluting stock solutions in a mixture of mQ H_2_O/ACN 1/1 (*v*/*v*) up to a concentration of 1.0 μg mL^–1^. The mass spectra of each analyte were acquired by introducing the working solution via syringe pump at 10/L min^–1^. Raw data were collected and processed by Varian Workstation, version 6.8, software. General synthetic scheme of compounds **3a**–**k** (Scheme 3).

A solution of **1** or **4** (equivalent to 0.15 g, 1.0, respectively) was treated with the appropriate primary or secondary amine (1.0 eq.) in ACN (5 mL) followed by addition of Et_3_N (1.1 eq.) The reaction mixture was refluxed o.n, then quenched with 1.0 M HCl aq. solution. Then, the obtained precipitate was filtered-off and washed with Et_2_O (3 × 5 mL) and dried under vacuum to obtain title compounds which were purified by silica gel column chromatography eluting with 10% MeOH/DCM *v*/*v*.

*N-(2-Hydroxy-5-sulfamoylphenyl)pyrrolidine-1-carboxamide* **3a**: 65% yield; δ_H_ (400 MHz, DMSO-*d*_6_) 1.92 (4H, m), 3.43 (4H, t, *J* 6.4), 6.97 (1H, d, *J* 8.4), 7.15 (2H, s, exchange with D_2_O, SO_2_N*H*_2_), 7.38 (1H, dd, *J* 2.1, 8.4), 7.72 (1H, s, exchange with D_2_O, N*H*), 8.19 (1H, d, *J* 2.1), 10.90 (1H, s, exchange with D_2_O, O*H*); δ_C_ (100 MHz, DMSO-*d*_6_) 26.0, 46.6, 116.4, 119.9, 122.2, 128.6, 135.7, 151.1, 154.9; *m*/*z* (ESI positive) [M+H]^+^.

*N-(2-Hydroxy-5-sulfamoylphenyl)piperidine-1-carboxamide* **3b**: 55% yield; Purified by flash chromatography by eluting with MeOH/DCM 7% *v*/*v*; δ_H_ (400 MHz, DMSO-*d*_6_) 1.54 (4H, m), 1.62 (2H, m), 3.47 (4H, m), 6.97 (1H, d, *J* 8.4), 7.14 (2H, s, exchange with D_2_O, SO_2_N*H*_2_), 7.40 (1H, dd, *J* 2.3, 8.4), 8.06 (2H, m, 1H exchange with D_2_O, N*H*), 10.70 (1H, s, exchange with D_2_O, O*H*); δ_C_ (100 MHz, DMSO-*d*_6_) 24.8, 26.3, 45.7, 116.5, 121.0, 122.6, 128.6, 135.6, 151.9, 155.9; *m*/*z* (ESI positive) [M+H]^+^.

*N-(2-Hydroxy-5-sulfamoylphenyl)morpholine-4-carboxamide* **3c**: 54% yield; Purified by flash chromatography by eluting with MeOH/DCM 10% *v*/*v*); δ_H_ (400 MHz, DMSO-*d*_6_) 3.47 (4H, t, *J* 5.0), 3.66 (4H, t, *J* 5.0), 6.97 (1H, d, *J* 8.4), 7.16 (2H, s, exchange with D_2_O, SO_2_N*H*_2_), 7.40 (1H, dd, *J* 2.4, 8.4), 8.06 (1H, exchange with D_2_O, N*H*), 8.08 (1H, d, *J* 2.4), 10.60 (1H, brs, exchange with D_2_O, O*H*); δ_C_ (100 MHz, DMSO-*d*_6_) 45.0, 66.9, 116.2, 121.3, 122.8, 128.3, 135.5, 152.3, 156.2; *m*/*z* (ESI positive) [M+H]^+^.

*4-Hydroxy-3-(3-(2-(piperidin-1-yl)ethyl)ureido)benzenesulfonamide* **3d**: 66% yield; δ_H_ (400 MHz, DMSO-*d*_6_) 1.39–1.58 (6H, m), 2.36–2.41 (6H, m), 3.23 (2H, q, *J* 6.2), 6.90–6.95 (2H, m, 1H exchange with D_2_O, N*H*), 7.08 H exchange wit(2H, s, exchange with D_2_O, SO_2_N*H*_2_), 7.25 (1H, dd, *J* 2.3, 8.4), 8.24 (1H, s, exchange with D_2_O, N*H*), 8.56 (1H, d, *J* 2.3), 10.73 (1H, s, exchange with D_2_O, O*H*); δ_C_ (100 MHz, DMSO-*d*_6_) 25.0, 26.4, 37.4, 55.0, 59.2, 114.6, 116.9, 120.1, 129.5, 135.5, 149.2, 156.1; *m*/*z* (ESI positive) [M+H]^+^.

*4-Hydroxy-3-(3-(2-morpholinoethyl)ureido)benzenesulfonamide* **3e**: 25% yield; purified by flash chromatography by eluting with MeOH/DCM 10% *v*/*v*; δ_H_ (400 MHz, DMSO-*d*_6_) 2.40–2.44 (6H, m), 3.25 (2H, q, *J* 6.1), 3.63 (4H, t, *J* 4.7), 6.91 (1H, d, *J* 8.4), 6.96 (1H, t, *J* 6.1, exchange with D_2_O, NH), 7.08 (2H, s, exchange with D_2_O, SO_2_N*H*_2_), 7.25 (1H, dd, *J* 2.3, 8.4), 8.22 (1H, s, exchange with D_2_O, N*H*), 8.56 (1H, d, *J* 2.3), 10.74 (1H, s, exchange with D_2_O, O*H*); δ_C_ (100 MHz, DMSO-*d*_6_) 37.1, 54.2, 58.9, 67.1, 114.6, 116.9, 120.1, 129.5, 135.6, 149.9, 156.1; *m*/*z* (ESI positive) [M+H]^+^.

*4-Ethyl-N-(2-hydroxy-5-sulfamoylphenyl)piperazine-1-carboxamide* **3f**: 62% yield; δ_H_ (400 MHz, DMSO-*d*_6_) 1.06 (3h, t, *J* 7.1), 2.38–2.43 (6H, m), 3.49 (4H, t, *J* 4.8), 6.96 (1H, d, *J* 8.5), 7.13 (2H, s, exchange with D_2_O, SO_2_N*H*_2_), 7.40 (1H, dd, *J* 2.2, 8.5), 8.06 (2H, m, 1H exhange with D_2_O, N*H*), 10.65 (1H, brs, exchange with D_2_O, OH); δ_C_ (100 MHz, DMSO-*d*_6_) 12.7, 44.6, 52.4, 52.9, 116.3, 121.1, 122.6, 128.3, 135.5, 152.0, 155.9; *m*/*z* (ESI positive) [M+H]^+^.

*4-Hydroxy-3-(3-(pyridin-4-ylmethyl)ureido)benzenesulfonamide* **3g**; 35% yield; purified by flash chromatography by eluting with MeOH/DCM 10% *v*/*v*; δ_H_ (400 MHz, DMSO-*d*_6_) 4.38 (2H, d, *J* 5.8), 6.92 (1H, d, *J* 8.4), 7.06 (2H, s, exchange with D_2_O, SO_2_N*H*_2_), 7.26 (1H, dd, *J* 2.3, 8.4), 7.33 (2H, d, *J* 4.4), 7.54 (1H, t, *J* 5.8, exchange with D_2_O, N*H*), 8.30 (1H, s, exchange with D_2_O, N*H*), 8.55 (2H, d, *J* 5.8), 8.59 (1H, d, *J* 2.3), 10.76 (1H, s, exchange with D_2_O, O*H*); δ_C_ (100 MHz, DMSO-*d*_6_) 42.7, 114.6, 116.8, 120.4, 122.9, 129.3, 135.7, 149.0, 150.3, 150.5, 156.2; *m*/*z* (ESI positive) [M+H]^+^.

*4-Hydroxy-3-(3-(2-(pyridin-2-yl)ethyl)ureido)benzenesulfonamide* **3h**: 85% yield; δ_H_ (400 MHz, DMSO-*d*_6_) 2.94 (2H, t, *J* 6.9), 3.52 (2H, q, *J* 6.9), 6.90 (1H, d, *J* 8.4), 7.00 (1H, t, *J* 6.9, exchange with D_2_O, N*H*), 7.08 (2H, s, exchange with D_2_O, SO_2_N*H*_2_), 7.24–7.28 (2H, m), 7.32 (1H, d, *J* 7.8), 7.75 (1H, dt, *J* 1.8, 7.8), 8.13 (1H, s, exchange with D_2_O, N*H*), 8.56 (1H, m), 8.59 (1H, d, *J* 2.3), 10.71 (1H, brs, exchange with D_2_O, O*H*); δ_C_ (100 MHz, DMSO-*d*_6_) 38.9, 39,7, 114.6, 116.8, 120.2, 122.4, 124.2, 129.5, 135.6, 137.4, 149.1, 150.0, 156.1, 160.1; *m*/*z* (ESI positive) [M+H]^+^.

*4-(2-(Cyclohexylamino)-2-oxoethyl)-N-(2-hydroxy-5-sulfamoylphenyl)piperazine-1-carboxamide* **3i**: 55% yield; purified by flash chromatography by eluting MeOH/DCM 10% *v*/*v*); δ_H_ (400 MHz, DMSO-*d*_6_) 1.15–1.36 (5H, m), 1.57–1.76 (5H, m), 2.50 (4H, t, *J* 4.8), 2.98 (2H, s), 3.21 (2H, d, *J* 4), 3.52 (4H, t, *J* 4.8), 3.62 (1H, m), 4.12 (1H, q, *J* 5.1), 6.97 (1H, d, *J* 8.4), 7.15 (2H, s, exchange with D_2_O, SO_2_N*H*_2_), 7.40 (1H, dd, *J* 2.3, 8.4), 7.56 (1H, d, *J* 8.3, exchange with D_2_O, N*H*), 8.06 (2H, m, 1H exchange with D_2_O, N*H*), 10.62 (1H, s, exchange with D_2_O, O*H*); δ_C_ (100 MHz, DMSO-*d*_6_) 25.5, 26.1, 33.3, 44.6, 48.0, 49.5, 53.3, 61.9, 116.3, 121.3, 122.7, 128.3, 135.6, 152.0, 155.9, 168.7; *m*/*z* (ESI positive) [M+H]^+^.

*4-Hydroxy-3-(3-(4-hydroxybutyl)ureido)benzenesulfonamide***3j**: 60% yield; δ_H_ (400 MHz, DMSO-d_6_) 1.49 (4H, m), 3.11 (2H, q, J 5.6), 3.44 (2H, q, J 5.6), 4.44 (1H, t, J 5.6, exchange with D_2_O, OH), 6.91 (1H, d, J 8.4), 6.95 (1H, t, J 5.6, exchange with D_2_O, NH), 7.07 (2H, s, exchange with D_2_O, SO_2_NH_2_), 7.25 (1H, dd, J 2.3, 8.4), 8.08 (1H, s, exchange with D_2_O, NH), 8.58 (1H, d, J 2.3), 10.71 (1H, s, exchange with D_2_O, OH); δ_C_ (100 MHz, DMSO-d_6_) 27.3, 30.8, 39.8, 61.4, 114.6, 116.7, 120.1, 129.6, 135.7, 148.9, 156.1; *m*/*z* (ESI negative) [M+H]^−^. 304.1

*4-(2-(3-(2-Hydroxyphenyl)ureido)ethyl)benzenesulfonamide***3k**: 70% yield; δ_H_ (400 MHz, DMSO-*d*_6_) 2.85 (2H, t, *J* 6.9), 3.40 (2H, q, *J* 6.9), 6.70–6.82 (3h, m), 6.90 (1H, t, *J* 6.9, exchange with D_2_O, N*H*), 7.32 (2H, s, exchange with D_2_O, SO_2_N*H*_2_), 7.47 (2H, d, *J* 8.2), 7.80 (2H, d, *J* 8.2), 7.91–7.94 (2H, m, 1H exchange with D_2_O, N*H*), 9.82 (1H, s, exchange with D_2_O, O*H*); δ_C_ (100 MHz, DMSO-*d*_6_) 36.4, 41.1, 115.6, 119.5, 120.0, 122.1, 126.6, 129.3, 130.1, 142.9, 144.7, 146.3, 156.4; *m*/*z* (ESI positive) [M+H]^+^.

### 3.2. Carbonic Anhydrase Inhibition

An Applied Photophysics stopped-flow instrument was used to assay the CA catalyzed CO_2_ hydration activity [14]. Phenol red (at a concentration of 0.2 mM) was used as an indicator, working at the absorbance maximum of 557 nm, with 20 mM Hepes (pH 7.4) as a buffer and 20 mM Na_2_SO_4_ (to maintain constant ionic strength), following the initial rates of the CA-catalyzed CO_2_ hydration reaction for a period of 10–100 s. The CO_2_ concentrations ranged from 1.7 to 17 mM for the determination of the kinetic parameters and inhibition constants. Enzyme concentrations ranged between 5–12 nM. For each inhibitor, at least six traces of the initial 5–10% of the reaction were used to determine the initial velocity. The uncatalyzed rates were determined in the same manner and subtracted from the total observed rates. Stock solutions of the inhibitor (0.1 mM) were prepared in distilled–deionized water and dilutions up to 0.01 nM were done thereafter with the assay buffer. Inhibitor and enzyme solutions were preincubated together for 15 min at room temperature prior to the assay to allow for the formation of the E–I complex. The inhibition constants were obtained by non-linear least-squares methods using PRISM 3 and the Cheng–Prusoff equation, as reported earlier, and represent the mean from at least three different determinations. All CA isoforms were recombinant proteins obtained in house, as reported earlier [1,12,13].

### 3.3. Carbonic Anhydrase IX-Mimic Design 

Carbonic Anhydrase IX-mimic (CA IX-mimic) was designed by site-directed mutagenesis of Carbonic Anhydrase II (CA II) by Genis et al., 2009, and additionally developed by Pinard et al., 2013 [15,16]. Mutations are primarily in the active site of CA II to create CA IX-mimic and they include A65S, N67Q, E69T, I91L, F131V, K170E, and L204A. These residues include a set (67, 91, 92, 131) that has been named the “selective pocket” due to its highly variable nature across isoforms of αhCAs [17]. This high variability from isoform to isoform makes this region ideal for targeting and detargeting different isoforms of CA via rational drug design. 

### 3.4. Protein Expression and Purification 

To express CA II and CA IX-mimic, cDNA in a pET expression vector was transformed into BL21(DE3) competent *E. coli* cells according to the manufacturers protocol. Cells were thawed on ice, gently mixed, and then pipetted into a 1.5 mL tube on ice. An amount of 100 ng of DNA are then added to the mixture and gently mixed before being incubated on ice for 30 min. At the end of the incubation, cells are heat shocked in a water bath at 42 °C for exactly 10 s before incubating on ice for 5 more minutes. An amount of 950 μL of Super Optimal broth with catabolite expression (SOC) media is then added to the mixture and then incubated for 1 h at 37 °C while shaking at 250 rpm. LB Agar plates are warmed to 37 °C and 50 μL of the cell mixture is spread onto the plate before overnight incubation [18].

BL21(DE3) competent cells containing the CA II expression vector are added to 1 L Luria broth media containing 1 mL of 100 mg/mL ampicillin and incubated with shaking at 37 °C until the optical density at 600 nm was at least 0.6. Protein expression was induced by the addition of 1 mL of 100 mg/mL Isopropyl-β-d-thiogalactopyranoside (IPTG) and proper protein folding was aided by adding 1 mL of 1 M zinc sulfate. The cells were incubated for 3 h and harvested by centrifugation at 4000 rpm, the supernatant was then decanted, and the pellet was frozen at −20 °C overnight. The following day, cell pellets were resuspended in 40 mL of Wash Buffer 1 (WB1, 0.2 M sodium sulfate, 0.1 M Tris–HCl, pH 9.0). A microfluidizer (LM10, Microfluidics, Boston, MA, USA) was used to lyse the cells before centrifugation at 15,000× *g* for 70 min at 4 °C to pellet debris. The supernatant was then filtered with a 0.8-μm filter (09-720-4, Fisherbrand, Boston, MA, USA). 

Proteins were then purified using a *para*-aminomethylbenzenesulfonamide agarose resin affinity column. The column was equilibrated prior by filtering through a column volume (CV, roughly 10–12 mL) of WB1. The lysate was then loaded onto the column and washed with WB1 and Wash Buffer 2 (WB2, 0.2 M sodium sulfate, 0.1 M Tris–HCl, pH 7.0) to elute non-specific proteins. Protein was next eluted off the column with 0.4 M sodium azide in 50 mM Tris at pH 7.8. The eluent was added to an Amicon (Ultra-15 centrifugal filter, Millipore Sigma, Darmstadt, Germany) filtration device with a 10 kDa molecular weight cutoff. An amount of 10 mL of storage buffer (50 mM Tris-HCl, pH 7.8, filtered) was then added to the filter, centrifuged at 6000× *g* for 15 min, then resuspended. This was repeated five times to completely remove the sodium azide [19].

Final protein concentration was quantified by measuring absorbance at 280 nm with an extinction coefficient of 54,800 and samples were separated on a 12% SDS-PAGE gel to confirm successful protein expression. Gels were stained with Coomassie dye and shaken for 1 h at 4 °C before alternating cycles of destaining and washing to clearly identify purified protein bands at 30 kD.

### 3.5. Crystal Preparation 

Crystals of CA II and CA IX-mimic were obtained using the hanging drop vapor diffusion method with a precipitant solution comprised of 1.6 M sodium-citrate, 50 mM Tris at a pH of 7.8. Purified protein was diluted to 10 mg/mL prior to crystallization with storage buffer. An amount of 5 μL total (2.5 μL each) of both the 10 mg/mL protein and precipitant solution was added in a 1:1 ratio to siliconized glass cover slips, inverted, suspended over a well containing 500 μL of the precipitant, and sealed shut with grease to ensure the system was closed. 

Crystal formation was observed within 24 h. Compounds were dissolved in 100% DMSO, then diluted 1:10 in the precipitant solution to a final concentration of approximately 7 mM. An amount of 1 µL of each compound was added to a crystal, for a final volume of 6 µL with approximately 1.67% DMSO by volume. Inhibitor–crystal solutions were incubated for 48 h before being mounted onto loops. 

### 3.6. Data Collection and Analysis

The soaked crystals were harvested with 150–200-micron loops, flash frozen in liquid nitrogen, and shipped to the Cornell High Energy Synchrotron Source (CHESS) and Stanford Synchrotron Radiation Lightsource (SSRL). X-ray diffraction data was collected on the F1 beamline at CHESS using a Pilatus 6M detector (Dectris, Philadelphia, PE, USA) with 0.5–1° oscillations, at a wavelength of 0.9900 to 1.000 Å, and a detector distance of 250–300 mm. Between 180 and 720 images were collected per loop-mounted crystal for 180°–360° of data. 

Data sets were indexed and integrated using XDS, then merged and scaled to space group P 1 2_1_ 1 using Aimless from the CCP4 suite [20,21]. Initial phase determination, coordinate refinement, and generation of the ligand restraint files were performed using phenix.phaser, phenix.ligandfit, and phenix.refine in the PHENIX software package and a modified coordinate file with the zinc removed of PDB entry 3kS3 as a search model for molecular replacement [22,23]. Inhibitor compounds were built into the model using phenix.ligandfit and Coot during refinement for manual adjustment and real space refinement [24]. Figures were generated using molecular graphics software, PyMol, and LigPlot+ (2.2.4, Cambridge, England), which was used to determine hydrogen bonding and hydrophobic interactions [25,26].

## 4. Conclusions

Herein, the strategic development of CAIs bearing conformationally restricted alkyl/aryl ureas, which are thus endowed with differential rotational features, is successfully applied to the obtainment of highly potent (i.e., sub-nanomolar) and selective ligands acting on CA IX. The binding modes of **3g**, **3h**, and **3j** were deeply examined at the atomic level by means of X-ray crystallographic experiments on their adducts with either CA II and IX-mimic isozymes. We are confident that this study validates our strategic design based on the ad hoc manipulation of key moieties (such as the ureido) in order to adapt their physical/chemical features to the biological target of interest (i.e., CA IX), and therefore it significatively advances the CA field that is strictly committed to the identification of the structural determinants governing the isoform selectivity in small molecules.

## Figures and Tables

**Table 1:** Inhibition data of human CA isoforms I, II, IX, and XII with derivatives **3a**–**k** and the standard sulfonamide inhibitor acetazolamide (**AAZ**) by the stopped-flow CO_2_ hydrase assay [14].

K_I_ * (nM)					
Compound	CAI	CA II	CA IX	CA XII	SI (IX/II)
**3a**	583.1	3182	369.1	91.3	0.12
**3b**	8654	914.5	307.9	506.6	0.34
**3c**	4000	8026	139.8	73.8	0.017
**3d**	688.6	287.8	4116	659.1	14.3
**3e**	9206	2995	2142	628.8	0.71
**3f**	8987	7741	2153	505.3	0.28
**3g** [10]	89.5	463	0.30	85.1	0.0006
**3h** [10]	571	331	0.24	65.9	0.0007
**3i**	697.9	405.4	204.2	33.7	0.50
**3k** [10]	798	280	215	47.1	0.77
**3j**	315.5	75.9	34.7	238.6	0.46
**AAZ**	250.0	12.1	25.8	5.7	2.1

* Mean from assays done in triplicate (errors were in the range of ±5–10% of the reported values).

**Table 2:** X-ray diffraction data and refinement statistics for compounds **3g**, **3h**, and **3j** in complex with CA II and CA IX-mimic. Statistics for the highest resolution shell are in parentheses.

Compound	3g		3h		3j	
Protein	CA II	CA IX-Mimic	CA II	CA IX-Mimic	CA II	CA IX-Mimic
Resolution Range (Å)	25.1-1.4 (1.5-1.4)	28.8-2.0 (2.1-2.0)	25.1-1.4 (1.5-1.4)	31.9-1.3 (1.4-1.3)	30.5-1.4 (1.5-1.4)	39.8-1.1 (1.2-1.1)
Space Group	P 1 2_1_ 1	P 1 2_1_ 1	P 1 2_1_ 1	P 1 2_1_ 1	P 1 2_1_ 1	P 1 2_1_ 1
Unit Cell: a, b, c (Å) α, β, γ (°)	42.0, 40.8, 71.1 90, 104.1, 90	42.5, 41.6, 72.5 90, 103.5, 90	42.0, 40.9, 71.4 90, 104.2, 90	41.9, 41.2, 71.9 90, 103.8, 90	42.3, 41.1, 71.9 90, 104.2, 90	42.2, 41.4, 72.2 90, 103.6, 90
Total Reflections	123,074 (11,706)	30,931 (3,110)	130,379 (12,291)	189,486 (17,491)	134,739 (12,839)	279,296 (19,725)
Redundancy	3.4 (3.3)	1.9 (1.9)	3.3 (3.3)	3.3 (3.1)	3.3 (3.3)	3.3 (2.9)
Completeness (%)	96 (93)	94 (97)	97 (93)	100 (98)	99 (96)	92 (71)
Mean I/σ (*I*)	17.4 (1.9)	7.6 (2.6)	15.2 (1.9)	14.7 (1.8)	18.6 (1.8)	10.9 (1.3)
R-Merge/R-Symm	0.04 (0.52)	0.08 (0.33)	0.04 (0.59)	0.04 (0.57)	0.04 (0.57)	0.05 (0.76)
R-Work	0.17 (0.30)	0.19 (0.27)	0.17 (0.27)	0.16 (0.29)	0.16 (0.41)	0.17 (0.24)
R-Free	0.19 (0.32)	0.25 (0.35)	0.19 (0.31)	0.18 (0.29)	0.18 (0.43)	0.17 (0.23)
No. of non-H atoms: CA, Inhibitor, water	2080, 33, 291	2042, 31, 36	2062, 24, 344	2061, 36, 408	2062, 29, 324	2061, 27, 208
Ramachandran Statistics: Favored, Allowed, Outliers (%)	97.3, 2.7, 0.0	96.1, 3.9, 0.0	96.9, 3.1, 0.0	96.1, 3.9, 0.0	97.6, 2.4, 0.0	96.9, 3.1, 0.0
Average B-factors: Overall, CA, Inhibitor, solvent (Å^2^)	23.0, 21.9, 30.2, 31.7	27.4, 27.2, 40.7, 26.3	21.2, 20.0, 17.7, 30.8	18.3, 16.3, 27.8, 29.8	19.8, 18.1, 31.3, 31.2	18.6, 17.1, 25.3, 31.7

## Data Availability

All six X-ray crystallography structures were deposited to the Protein Data Bank (PDB) under deposition IDs given in the discussion. This data can be accessed from the PDB at https://www.rcsb.org/ (accessed on 6 January 2023).
